# Study of water adsorption on graphene edges

**DOI:** 10.1039/c8ra00002f

**Published:** 2018-03-21

**Authors:** Lijuan Jiang, Jinlong Wang, Peng Liu, Wei Song, Bingling He

**Affiliations:** Department of Physics and Electronic Engineering, Xinxiang University Xinxiang 453003 China; State Key Laboratory of Low-Dimensional Quantum Physics, Department of Physics, Tsinghua-Foxconn Nanotechnology Research Center, Tsinghua University Beijing 100084 China pengliu@mail.tsinghua.edu.cn

## Abstract

Water adsorption on graphene edges was studied by field emission (FE) experiments and first principles simulation. By analyzing the FE current change with temperature, it was concluded that the intrinsic FE of a graphene edge is consistent with Fowler–Nordheim (FN) theory. The noise of *IV* and non-linearity of FN curves at room-temperature can be interpreted by the adsorption effects. Water is speculated as the most responsible gas specie. We have calculated the work function of graphene by VASP. The results show that water adsorption will lower the work function of the graphene edge, while increasing the work function of the graphene surface.

## Abstract 2

Water adsorption on graphene edges was studied by field emission (FE) experiments and first principles simulation.

## Graphical abstract

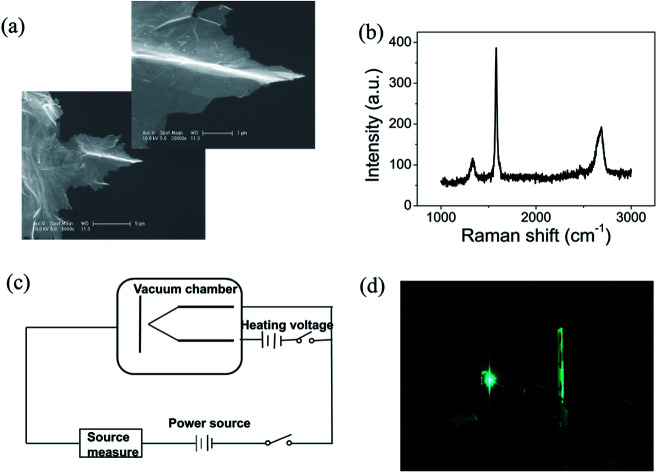

## 1. Introduction

Graphene, a two-dimensional (2D) single layer of carbon atoms bonded in a hexagonal lattice,^1,2^ has attracted tremendous attention due to its excellent optical transparency,^3^ high electrical conductivity,^4^ mechanical flexibility,^3,5^ and strong thermal/chemical stability.^6–9^ Earlier studies have focused on characterizing the unusual electronic and transport properties of graphene, particularly as a massless Dirac Fermion system.^4,10,11^ Of the many theoretical and experimental studies of graphene, a substantial portion are devoted to the properties of graphene edges,^2,11–13^ whose electronic states are obviously influenced by the local geometric structure and show many unique characteristics.^13–15^ The adsorption at an edge can also alter their properties greatly. Small gas molecules such as H_2_O, H_2_, O_2_, CO, NO_2_, and NO, will influence the electronic structure and other properties of graphene,^16–26^ the adsorption of some metal atoms such as lithium^27^ potassium^28^ have been calculated. However, there is no convenient and efficient experimental method to study the adsorption on graphene edge. Graphene edge show significant field emission (FE) ability,^29–32^ which can be a very suitable and simple method to study the adsorption on nanomaterial. We have studied the water adsorption on multiwalled carbon nanotube by analyzing the FE behavior at different temperature.^33^ In this paper, we have studied water adsorption on graphene edge by FE method. It has been found that the water adsorption will lower the work function of graphene edge. The first principle calculation based on VASP further verified the experimental results.

## 2. Experimental results and discussion

**Fig. 1:**
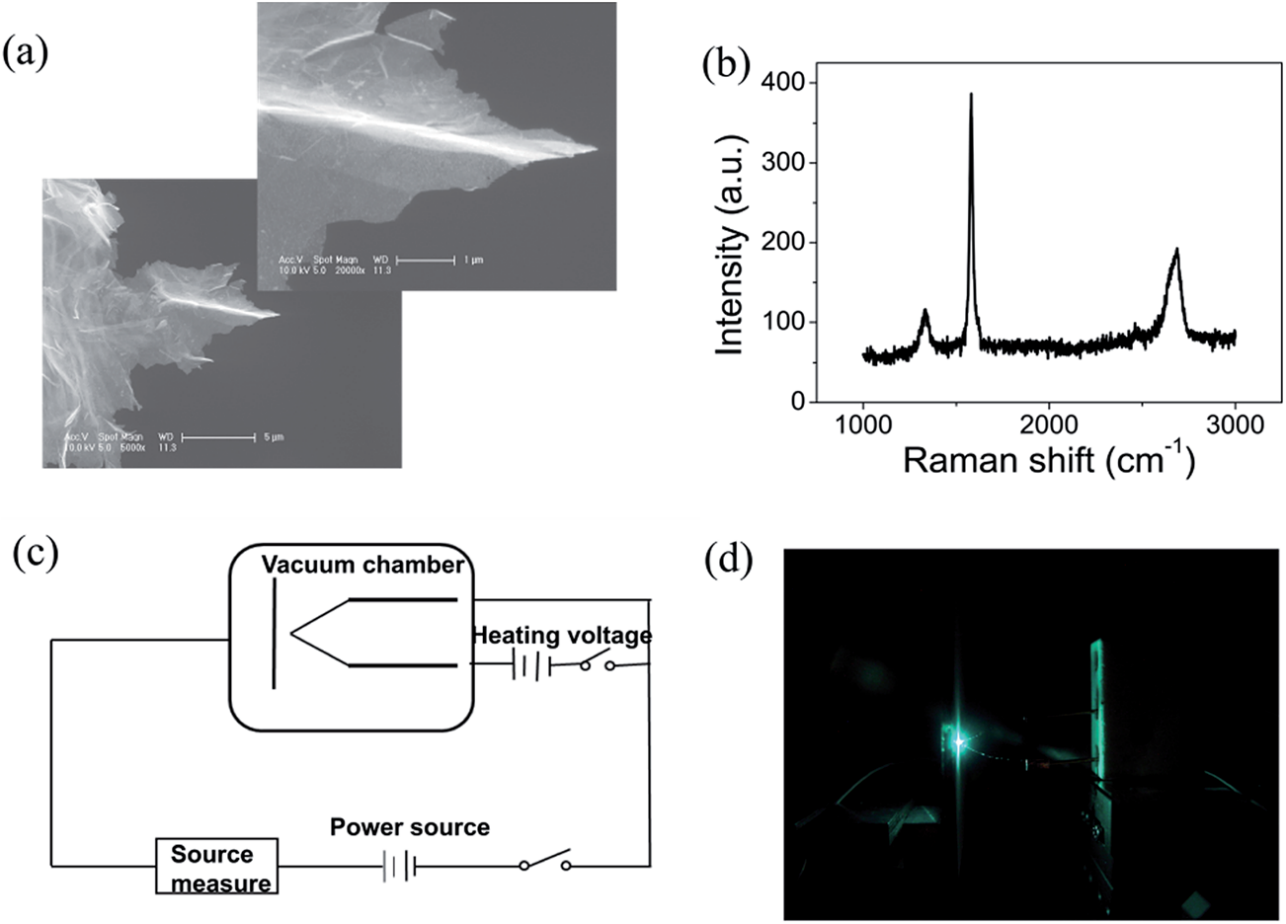
(a) The scanning electron microscope (SEM) images of the graphene. The left bottom is at 5000×, the right upper is at 20 000×. (b) Raman spectrum of graphene excited by 514 nm laser. (c) Schematic diagram of the circuit. (d) Photo of the phosphor anode lighted by the FE of graphene.

Graphene were prepared by the chemical method.^34^ Scanning electron microscopy (SEM) and Raman spectroscopy indicate that the sample is few-layer graphene. Fig. 1(a) shows a SEM image of graphene. We can find that graphene have numerous thin open edges and tips. The Raman spectrum excited by a 514 nm laser is shown in Fig. 1(b), the high 2D peak verifies that the sample is composed predominately by few layer graphene.^35^ The graphene field emitter is fabricated by sticking them on a molybdenum wire of 0.1 mm diameter with organic binder, which can be thoroughly evaporated after being heated at 350 °C in air a few minutes. The FE experiment was conducted in a high-vacuum chamber with a base pressure of 10^−5^ Pa. Fig. 1(c) shows the schematic diagram of the circuit. Two metal rod electrodes were used to heat the graphene with current in the vacuum chamber. Keithley 237 high voltage source measure unit was used to supply the electric voltage, an indium tin oxide (ITO) glass anode coated with phosphor was used as anode. The distance between the anode and cathode is about 200 μm. Fig. 1(d) is a photo of the phosphor anode lighted by the FE of graphene cathode at 1100 V.

**Fig. 2:**
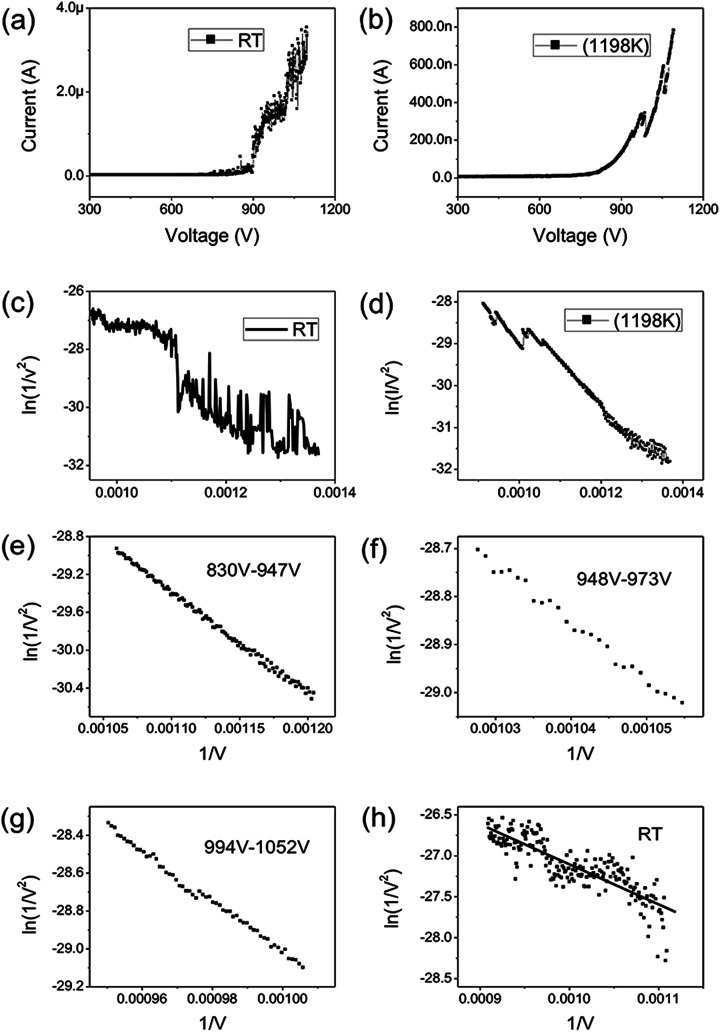
(a) FE *IV* curves of graphene at RT. (b) FE *IV* curves of graphene at 1198 K. (c) FN curves at RT. (d) FN curves at 1198 K. (e) FN curves in the range of 830–947 V at 1198 K. (f) FN curves in the range of 948–973 V at 1198 K. (g) FN curves in the range of 994 V–1052 V at 1198 K. (h) FN curves in the range of 900–1100 V at RT.

Fig. 2(a) shows the *IV* curve of the graphene at room temperature (RT). Here a DC voltage sweeping from 300 V to 1100 V was applied to the sample. It can be seen that the curves show obvious noise. The emission current is 0.2 μA at 3.75 V μm^−1^ and 4 μA at 5.5 V μm^−1^. Fig. 2(b) shows the *IV* curve of the graphene at 1198 K. It can be seen that the *IV* curve is very smooth. Fig. 2(c) and (d) display the corresponding FN plots. The plot at RT show obvious noise and the plot at 1198 K is a smooth and straight line of multi-segment. Comparing the noise curves in Fig. 2(a) and (c) and the smooth curves in Fig. 2(b) and (d), we can know that the noise is related to the temperature. The same as previous reports,^36^ heating can be used as a useful method to remove adsorbents. The dwelling time of the adsorbents will be shortened at high temperature. Therefore, the FE at 1198 K in this experiment is determined as the intrinsic FE of graphene after the removal of adsorbents (the different segments of FN plot at 1198 K are caused by a small number of samples being detached during emission).

According to the FN equation,^37^1
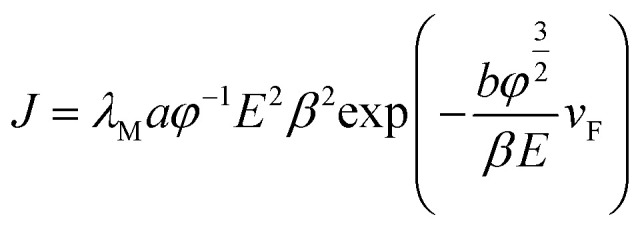
where, *J* is the current density, *λ*_M_ a macroscopic pre-exponential correction factor, *a* and *b* are constants (*a* = 1.54 × 10^−6^ A eV V^−2^, *b* = 6.83 eV^−3/2^ V nm^−1^), *ϕ* is the work function of the emitter (4.74 eV for graphene^33^), *E* is the applied electric field^38^ (*E* = *V*/*d*, where *V* is the voltage applied between the flat cathode and the anode screen and distheir separation). *β* is the local electrical field enhancement factor, *v*_F_ (correction factor) is a value of the Schottky–Nordheim barrier function *v*. The field enhancement factor *β* can be calculated from the slope ‘*m*’ of the FN curves (the curves of ln(1/*V*^2^) *versus* 1/*V*), using the following equation.2
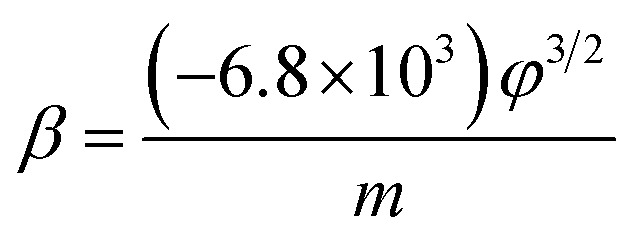


The field-enhancement factor (*β*) of graphene was calculated and listed in Table 1. The *β* of graphene was 13 063.81424, 11 725.88950, 10 676.06139 at 830V–947 V, 948–973 V, 994–1052 V. There is no big difference for the *β* of three segments, which is also close to the aspect ratio estimation based on the SEM image shown in Fig. 1(a). The large *β* verified that the FE comes from graphene edge. Because adsorption can not change geometric structure of graphene, the *β* at RT can be viewed as almost same as that at high temperature. The work function at RT calculated was 3.29 through solving eqn (2) based on *β* and the slope of FN plot at RT. The substituted *β* was the average value of three segments at high temperature. The work function at RT was small than this calculated by the Zhu F.^33^ This implies that adsorption can lower the work function of graphene edge.

**Table 1:** The intercept, slope and *β* of graphene at 1198 K and RT

	830–947 V	948–973 V	994–1052 V	RT
Intercept	−17.56889	−16.40583	−15.86982	−22.26244
Slope	−10743.26837	−11969.07597	−13146.05238	−4851.04459
*β*	13 063.81424	11 725.88950	10 676.06139	11 821.92171

**Fig. 3:**
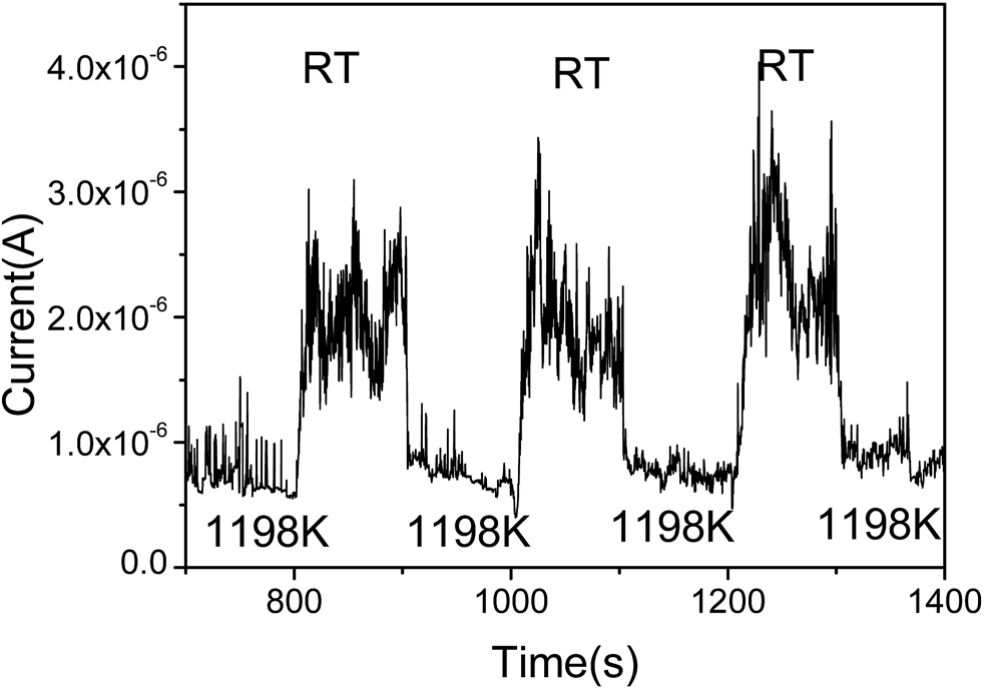
FE current switches between RT and 1198 K.

To further prove the influence of adsorption on the graphene, the FE current changes as the temperature switches between RT and 1198 K is shown in Fig. 3. It can be seen that the FE current decreases after heating to the higher temperature 1198 K, and the FE current will increase after stopping the heating. It indicates that the work function with adsorption is smaller than that at RT. It also shows that the noise of the FE current during heating is significantly lower than that at RT. We select the time range from 1018 s to 1107 s, the noise of the FE current without heating was calculated to be about 22.8%. At the time range from 1111 s to 1154 s, the noise of the FE current at high temperature was calculated to be about 8.99%, this further evidence that the FE of the graphene at RT is influenced by the adsorption.^36,39^

**Fig. 4:**
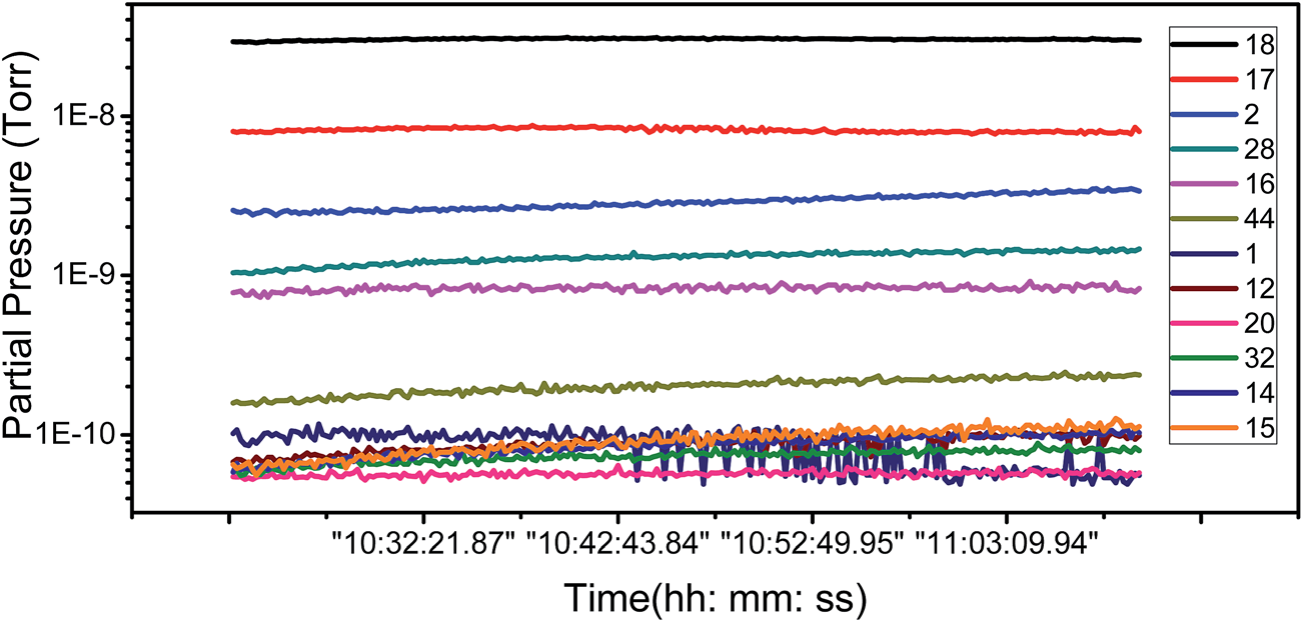
Partial pressure as a function of time for the 12 main residual gases species.

To ascertain the kind of adsorbates which have influence the FE of graphene, we have carried out an analysis of the adsorbent species of residual gas spectra in the vacuum chamber. Fig. 4 shows the 12 main species in the vacuum chamber. It can be seen that H_2_O, H_2_, and N_2_ are the most principal species. H_2_ and N_2_ are nonpolar molecules and deemed inactive, while H_2_O is a polar, active molecule and has a large proportion in the residual gas, so H_2_O is the most likely species to influence the adsorption of graphene.

## 3. First principle calculation of water adsorption on graphene

**Fig. 5:**
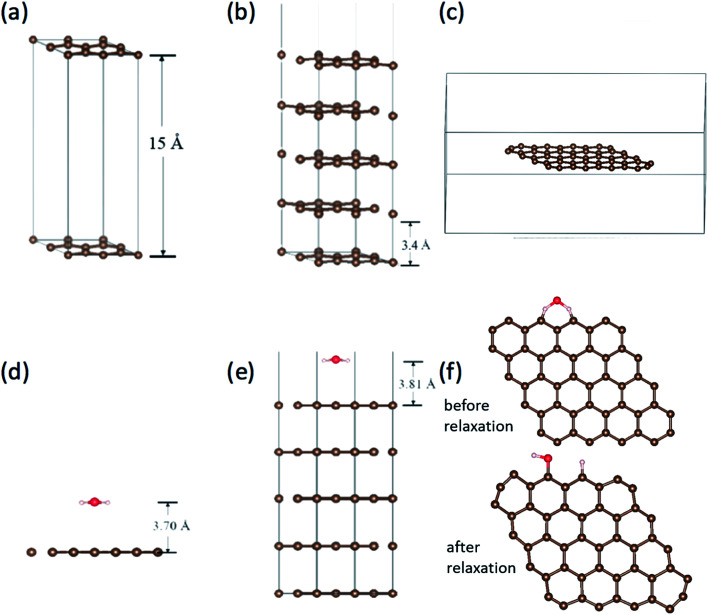
(a) The single layer graphene (2 × 2) surface slab model. (b) The five layer graphene (2 × 2) surface slab model, in which the vacuum layer doesn't shown overall. (c) The graphene in the cubic. The adsorption of H_2_O on (d) graphene surface, (e) graphite surface with five-layer graphene and (f) at the edge of graphene before and after relaxation.

Work function is the most direct parameter which can reflect the influence of the adsorption. We have calculated the work function of H_2_O/graphene systems with the projected augmented wave (PAW)^40^ formalism of DFT, as implemented in the VASP.^41^ The adopted exchange–correlation functional is the generalized gradient approximation with the Perdew–Burke–Ernzerhof (PBE).^42^ The cutoff energy for the plane wave basis set was taken as 400 eV. The geometry optimization of each isomer was carried out till the energy was converged to an accuracy of 10^−4^ eV. Brillouin zone integration is performed using Monkhorst–Pack grids^43^ for all calculations. We build three kinds of models as shown in Fig. 5(a–c), the single layer graphene (2 × 2) surface slab containing 15 Å vacuum layer in Fig. 5(a), five layer graphene (2 × 2) surface slab containing 15 Å vacuum layer to simulate the graphite surface in Fig. 5(b), the graphene fragment in the cubic with a dimension of 25 × 25 × 10 Å^3^ in Fig. 5(c). The *K*-point sampling is chosen as 5 × 5 × 1 and 1 × 1 × 1 for the slab and the cubic, respectively.

We calculated the adsorption of H_2_O in three kinds of models as shown in Fig. 5(d)–(f). It is found that there is no chemical adsorption between H_2_O and the graphene as shown in Fig. 5(d). The distance between H_2_O and the graphene surface up to 3.70 Å after relaxation. The interaction between them is only about 0.18 eV. In order to affirm our results, we simulate the graphite surface with five-layer graphene as shown in Fig. 5(e). The results are similar to previous one. The distance between H_2_O and the surface of graphite is up to 3.81 Å after relaxation. The interaction between them is 0.23 eV. Since the FE and adsorption/desorption happened on graphene edge in our experiment, we simulate the adsorption of H_2_O on the edge of graphene as shown in Fig. 5(f). It is found that the H atom and hydroxyl dissociate directly from H_2_O and adsorb at two zig–zag carbon atoms after relaxation. The adsorption energy of H and hydrogen bond is up to 5.93 eV, which is a strong interaction. Obviously, it is physical adsorptions for H_2_O on the surface of graphene and graphite, and it is chemical adsorption for H_2_O dissociation on the edge of graphene.

The work functions results are tabulated in Table 2. The work function without H_2_O are very close to the calculated work function using the average electrostatic potential of 7 × 7 pure grapheme^44^ and the experimentally measured work function 4.45–4.74 eV.^33,45–47^ It can be found that work function for graphene slabe surface increase from 4.24 eV to 5.15 eV due to H_2_O adsorption, work function for 5-layer graphene increase from 5.00 eV to 5.09 eV due to H_2_O adsorption, while work function for graphene edge decrease from 5.73 eV to 5.63 eV due to H_2_O adsorption. This is in good accordance with our experimental results that the FE comes from graphene edge and the FE current change with the adsorption/desorption.

**Table 2:** The work function of graphene adsorption and without H_2_O in the three kinds of models

Work function	Graphene slabe	5-Layer graphene	Graphene in the cubic
Without H_2_O	4.24	5.00	5.73
Exist H_2_O	5.15	5.09	5.63

## 4. Conclusions

In summary, the water adsorption on graphene edge has been studied. It was found that the adsorption induced the deviation from the FN model at RT. After removing the adsorbates by heating, the intrinsic FE of graphene edge follows the FN model very well. Water is speculated as the most responsible gas specie. The work function of graphene with H_2_O has calculated by VASP. The calculated results show that the water adsorption will lower the work function of graphene edge, while increase the work function of graphene surface. This is in good accordance with the experimental results.

## Conflicts of interest

There are no conflicts to declare.

## Supplementary Material
